# Variovorax arabinosiphilus sp. nov., Variovorax flavidus sp. nov., Variovorax gracilis sp. nov., Variovorax brevis sp. nov., Variovorax jilinensis sp. nov., Variovorax davisae sp. nov., Variovorax saccharolyticus sp. nov. and Variovorax fucosicus sp. nov., isolated from ginseng rhizosphere

**DOI:** 10.1099/ijsem.0.006895

**Published:** 2025-08-29

**Authors:** Yu-Hang Jiang, Ce-Ce Yin, Lei-Lei Yang, Yu-Hua Xin, Qing Liu, Jian Ye

**Affiliations:** 1 State Key Laboratory of Microbial Diversity and Innovative Utilization, Institute of Microbiology, Chinese Academy of Sciences, Beijing 100101, PR China; 2 Laboratory of Vector-Borne Diseases and Biomanufacturing, Institute of Microbiology, Chinese Academy of Sciences, Beijing 100101, PR China; 3 University of Chinese Academy of Sciences, Beijing 100049, PR China; 4 Guangxi University, Nanning, 530004, PR China; 5 China General Microbiological Culture Collection Center (CGMCC), Institute of Microbiology, Chinese Academy of Sciences, Beijing 100101, PR China

**Keywords:** ginseng, rhizosphere, *Variovorax*

## Abstract

Thirteen novel strains – J2L1-78^T^, J2P1-59^T^, J22R24^T^, J22R133^T^, J22P168^T^, J22P271^T^, J22R187^T^, J22R193^T^, J2L1-63, J2R1-6, J22G21, J22G47, and J31P216 – were isolated from the rhizosphere of 20-year-old ginseng in Jilin Province, P.R. China. These strains were Gram-stain-negative, aerobic and rod-shaped. Phylogenetic analysis based on the 16S rRNA gene sequences indicated their affiliation to the genus *Variovorax*, with 98.8–99.6% sequence similarity to *Variovorax ginsengisoli* Gsoil 3165^T^, *Variovorax ureilyticus* UCM-2^T^ and *Variovorax humicola* UC38^T^. Phylogenomic analysis demonstrated their distinctiveness from closely related species. Average nucleotide identity (ANI) values among these strains confirm their representation of eight distinct species; additionally, ANI and digital DNA–DNA hybridization values between these strains and their closest relatives were below 92.5 and 54.1%, respectively. Eight representative strains contained C_16 : 0_, summed feature 3 (C_16 : 1_* ω*7*c* and/or C_16 : 1_* ω*6*c*) and C_17 : 0_ cyclo as major fatty acids. Based on phenotypic, phylogenetic and genotypic data, these 13 strains comprise 8 novel species within the genus *Variovorax*, for which we propose the following names: *Variovorax arabinosiphilus* sp. nov. (J2L1-78^T^=CGMCC 1.60704^T^=KACC 23365^T^), *Variovorax flavidus* sp. nov. (J2P1-59^T^=CGMCC 1.60707^T^=KACC 23366^T^), *Variovorax gracilis* sp. nov. (J22R24^T^=CGMCC 1.61001^T^=KACC 23367^T^), *Variovorax brevis* sp. nov. (J22R133^T^=CGMCC 1.61263^T^=KACC 23368^T^), *Variovorax jilinensis* sp. nov. (J22P168^T^=CGMCC 1.64555^T^=KACC 23372^T^), *Variovorax davisae* sp. nov. (J22P271^T^=CGMCC 1.64593^T^=KACC 23373^T^), *Variovorax saccharolyticus* sp. nov. (J22R187^T^=CGMCC 1.64629^T^=KACC 23374^T^) and *Variovorax fucosicus* sp. nov. (J22R193^T^=CGMCC 1.64631^T^=KACC 23375^T^).

The genus *Variovorax*, within the family *Comamonadaceae* and order *Burkholderiales*, was established through the reclassification of *Alcaligenes paradoxus* as *Variovorax paradoxus* by Willems *et al*. [1]. *Variovorax* is widely recognized for its plant growth-promoting properties, particularly through the production of 1-aminocyclopropane-1-carboxylate (ACC) deaminase and auxin [2,4]. *Variovorax* plays a crucial role in mediating the bacteria-plant communication networks, effectively mitigating root growth inhibition caused by various bacterial strains, thus serving as a critical component of the plant root microbiome [5]. As of this writing, 13 *Variovorax* species have been validly published [6]. Most were isolated from soil [1,7,12], with fewer from the rhizosphere [2], sewage [13] or root [14]. Recently, we isolated thousands of bacterial strains from the rhizosphere soil of ginseng in Northeast China, identifying 13 *Variovorax* strains via 16S rRNA gene sequencing. Through a polyphasic approach, we proposed these strains as eight novel species and characterized their physiological traits. This study increases the *Variovorax* species count to 21, enhancing our understanding of its diversity within the plant root microbiome.

## Isolation and ecology

Rhizosphere soil samples were collected from 20-year-old ginseng in Tonghua City, Jilin Province, P.R. China, on 9 November 2022. Samples were homogenized, serially diluted with sterile water, and a 0.2 ml aliquot of a 10^−4^ dilution was spread onto peptone, yeast extract and glucose (PYG) [15] and Reasoner’s 2A (R2A) agar plates. After a 2-week incubation at 25 °C, single colonies were purified via repeated streaking on PYG or R2A agar. Thirteen strains – designated as J2L1-78^T^, J2P1-59^T^, J22R24^T^, J22R133^T^, J22P168^T^, J22P271^T^, J22R187^T^, J22R193^T^, J2L1-63, J2R1-6, J22G21, J22G47 and J31P216 – were obtained, routinely cultured on R2A agar at 28℃ and preserved in 10% (v/v) glycerol suspensions under liquid nitrogen storage.

## 16S rRNA phylogeny

Genomic DNA from the eight strains was extracted using the TaKaRa MiniBEST Bacteria Genomic DNA Extraction Kit version 3.0 (TaKaRa, Dalian, China) according to the manufacturer’s instructions. The 16S rRNA gene was amplified and sequenced with universal primers 27F and 1492R [16]. Closest phylogenetic neighbours were identified by comparing 16S rRNA gene sequences of the strains with those available in the EzBioCloud database [17]. Multiple sequence alignment was performed using MAFFT software (version 7.5) [18], and phylogenetic trees were constructed using the neighbour-joining (NJ), maximum-likelihood (ML) and maximum-parsimony (MP) algorithms in mega 12 with 1,000 bootstrap replicates [19]. Genetic distances for the NJ analysis were calculated using Kimura’s two-parameter model.

**Fig. 1.**
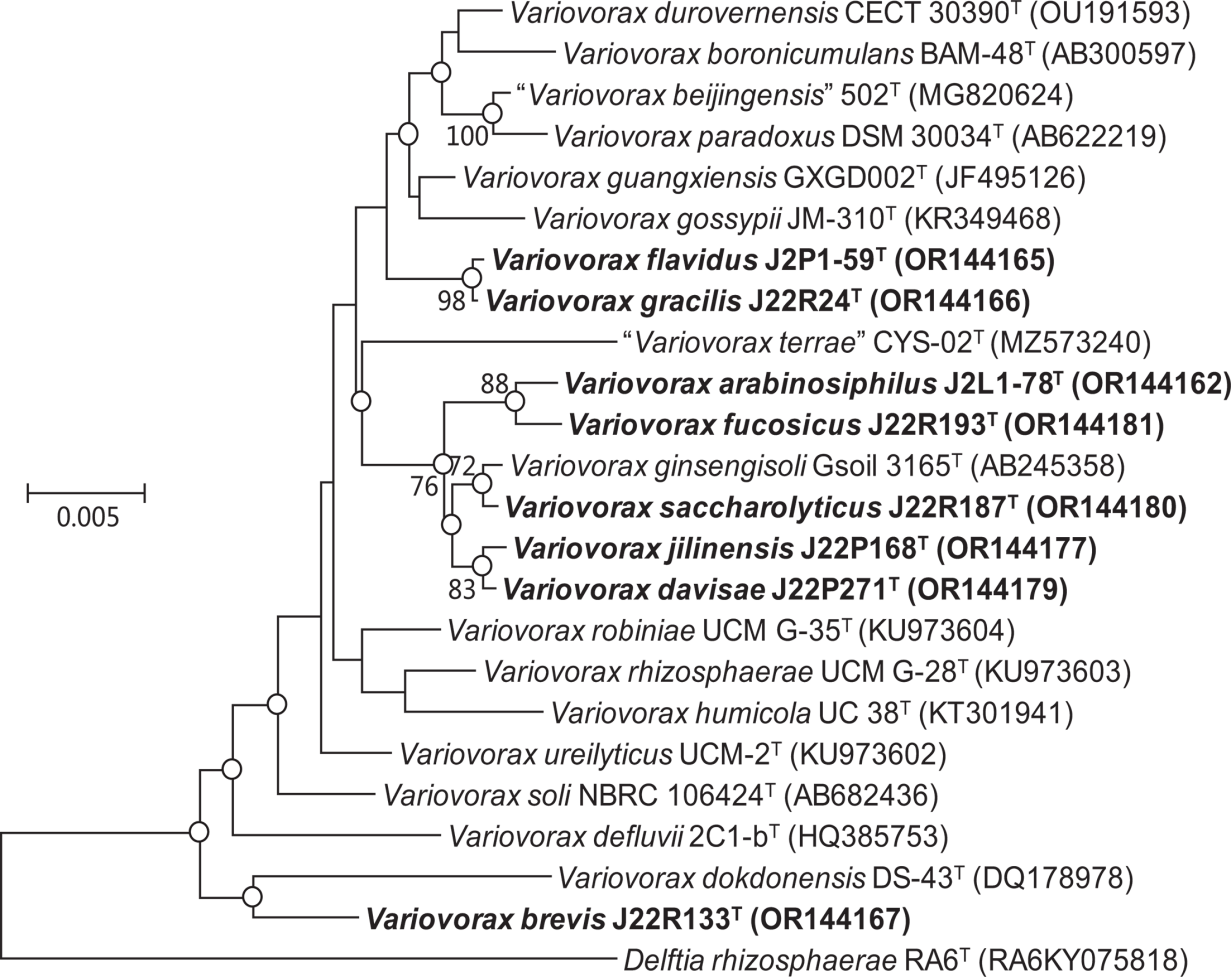
Phylogenetic tree of strains J2L1-78^T^, J2P1-59^T^, J22R24^T^, J22R133^T^, J22P168^T^, J22P271^T^, J22R187^T^, J22R193^T^ and related taxa based on 16S rRNA gene sequence comparisons using the NJ method. GenBank accession numbers are provided in parentheses. Open circles indicate that the corresponding branches were also recovered in the ML and MP trees. Bootstrap values (>50%) from 1,000 replicates are shown at branch nodes. Scale bar, 0.005 substitutions per nucleotide position.

Sequence comparisons confirmed these strains’ placement within the genus *Variovorax*, exhibiting 98.8–99.6% similarity to *Variovorax ginsengisoli* Gsoil 3165^T^, *Variovorax ureilyticus* UCM-2^T^ and *Variovorax humicola* UC38^T^ (Table S1, available in the online Supplementary Material). The 16S RNA gene sequences of the three strains, J2L1-78^T^, J2L1-63 and J2R1-6, are identical, with 100% similarity. Similarly, the sequences of strains J22R187^T^ and J31P216 also share 100% similarity. Additionally, the 16S RNA gene sequences of the three strains, J22R193^T^, J22G21 and J22G47, are completely identical, with a similarity of 100.0%. Therefore, strains J2L1-78^T^, J22R187^T^ and J22R193^T^ and other five strains were selected for phylogenetic analysis. The eight strains were distributed across multiple branches of the phylogenetic tree (Fig. 1). Strains J2P1-59^T^ and J22R24^T^ clustered together, forming a minor branch with 99.9% similarity. Strains J2L1-78^T^, J22R193^T^, J22R187^T^, J22P168^T^, J22P271^T^ and *V. ginsengisoli* Gsoil 3165^T^ grouped into another branch, all originating from ginseng field soil, with similarities ranging from 99.1% to 99.9%. Conversely, strain J22R133^T^ formed an independent branch, phylogenetically distant from other strains, showing 98.6% similarity to strain J2P1-59^T^. Trees constructed using ML and MP methods corroborated the NJ tree topology (Fig. 1).

## Genome features

Genome sequencing was performed on the Illumina HiSeq 4000 platform (Illumina, San Diego, CA, USA) following the manufacturer’s protocols for 150 bp paired-end reads. High-quality reads were assembled with the SPAdes program V3.15 [20], and assembly quality was evaluated using QUAST v5.0.2 [21]. Genome completeness was assessed with CheckM v1.1.6 [22], and annotation was conducted using Bakta v1.7.0 [23]. The genomic sequences generated in this study were compared with the available genomic data in the NCBI Datasets using FastANI v1.33 [24] to identify their closest relatives. In addition, genomic sequences representing novel *Variovorax* species were also selected from the GTDB Release R220 database [25] for phylogenetic tree construction, alongside species with validly published names. Eighty-one core genes were extracted from the whole-genome sequences via the UBCG 2 pipeline with default parameters [26]. Concatenated gene sequences were aligned using MAFFT software [18], and the optimal nucleotide substitution model was determined by IQTREE 2 [27]. A phylogenomic tree was constructed using the ML method under the GTR+F+I+R5 model with 1,000 bootstrap replicates. Average nucleotide identity (ANI) was calculated with FastANI v1.33 [24], and clustering of the pairwise symmetric dissimilarity matrix was performed using the ‘bactaxR’ package in R with average linkage hierarchical clustering method [28]. Digital DNA–DNA hybridization (dDDH) values were computed using the Type (Strain) Genome Server [29].

Genomic sequencing yielded high-quality assemblies for all 13 strains (Tables S2 and S3). Genome completeness achieved 99.5–100%, with contamination levels below 2.38%, indicating robust sequencing and assembly. Complete 16S rRNA gene sequences retrieved from their genomic sequence were identical to the corresponding PCR products. Genome sizes varied from 5.46 Mb (J22P168^T^) to 8.54 Mb (J22R133^T^), with contig counts between 9 and 204, and G+C content between 65.4 and 68.2 mol%. No CRISPR arrays were detected, suggesting limited phage immunity. Each strain contained 6–14 ncRNAs, 43–53 tRNAs, 3–6 rRNAs and 1 tmRNA. Hypothetical proteins (282–946) and pseudogenes (2–11) varied, indicating potential functional diversity. Annotated CDS ranged from 5,126 to 7,867. Notably, the *acdS* gene, which encodes ACC deaminase and functions in ACC degradation, was present in strains J22R193^T^, J2L1-78^T^, J22R24^T^, J22R187^T^, J2L1-63, J2R1-6, J22G21, J22G47 and J31P216. Additionally, the *aldA* gene, encoding aldehyde dehydrogenase subunit A and involved in indole-3-acetic acid (IAA) synthesis, was identified in strains J2P1-59^T^, J22R24^T^, J22P271^T^, J22R187^T^, J31P216 and J22R133^T^. These findings suggest the plant growth-promoting potential of these strains.

A total of 338 *Variovorax*-assigned genomic sequences, comprising 224 isolates and 114 metagenome-assembled genomes (MAGs), were retrieved from NCBI Datasets for comparative analysis. No genomic sequences exceeded a 95% ANI value with the 13 study strains, indicating their novelty within the genus *Variovorax* and absence from both cultured isolates and MAGs. ANI values among J2L1-78^T^, J2L1-63 and J2R1-6 were 100%, while J22R187^T^ and J31P216 shared 98.8%, and J22R193^T^, J22G21 and J22G47 ranged from 98.4% to 100.0%, suggesting that these eight strains represent three distinct species (Table S4). ANI values between these eight strains and the remaining five strains range from 81.8% to 90.9% (Table S4). Eight representative strains (J2L1-78^T^, J2P1-59^T^, J22R24^T^, J22R133^T^, J22P168^T^, J22P271^T^, J22R187^T^ and J22R193^T^) were selected for further analysis. Pairwise ANI values among these eight strains and 15 related species revealed a maximum of 94.9% between ‘*Variovorax beijingensis*’ 502^T^ and *V. paradoxus* NBRC 15149^T^ and 92.5% between J22P271^T^ and *V. ginsengisoli* CGMCC 1.12090^T^. All other ANI values between the eight strains and their close relatives were below 92.5%, supporting their classification as distinct species [30]. The highest dDDH value (59.0%) was observed between ‘*Variovorax beijingensis*’ 502^T^ and *V. paradoxus* NBRC 15149^T^ with other values ranging from 20.8% to 54.1%, well below the 70% bacterial species threshold [31]. ANI clustering (Fig. S1) and Genome blast Distance Phylogeny (Fig. S2) further confirmed these eight strains as eight novel species within the genus *Variovorax*.

**Fig. 2.**
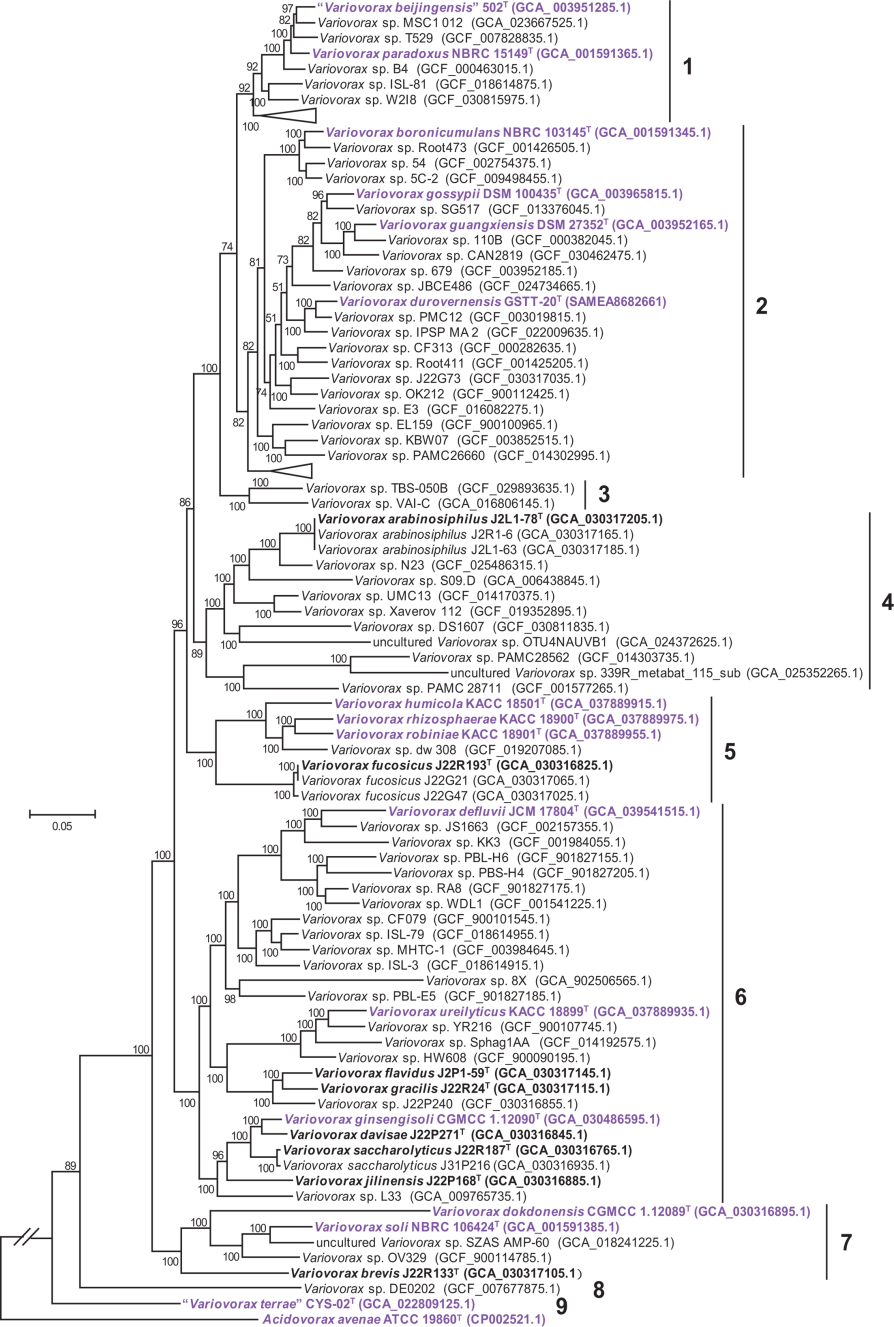
Phylogenetic tree of 13 study strains and related taxa, inferred using the ML method with the GTR+F+I+R5 model, based on a concatenated alignment of 81 core genes. Bootstrap values (>50%) from 1,000 replicates are shown at branch nodes. The purple and bold letters represent published species and novel species described in this study, respectively. Scale bar, 0.05 substitutions per nucleotide position.

In addition to the 13 study strains and all 15 type strains, 76 genomic sequences representing putative novel *Variovorax* species were selected from the GTDB Release R220 database for phylogenomic analysis (Fig. 2). Pairwise ANI analysis revealed 99 distinct species within the tree, expanding the known *Variovorax* diversity from 15 species. The strains formed nine clades based on phylogenetic affinity. Clusters 3, 8 and 9 contained 1 or 2 species each, suggesting limited diversity under exploration. Species with validly published names were distributed across clusters 1, 2, 5, 6 and 7, while cluster 4, with 10 species (including two MAG-derived), lacked known species. Strain J2L1-78^T^ represented the first type strain of a novel species in cluster 4. Strain J22R193^T^ clustered with *V. humicola* KACC 18501^T^, *Variovorax rhizosphaerae* KACC 18900^T^, *Variovorax robiniae* KACC 18901^T^ and *Variovorax* sp. dw 308 in cluster 5. The remaining strains (J2P1-59^T^, J22R24^T^, J22P271^T^, J22R187^T^ and J22P168^T^), representing five novel species, were predominantly located in cluster 6. Strain J22R133^T^ was positioned within cluster 7 and constituted a distinct branch.

## Physiology and chemotaxonomy

Colony morphology was examined after 3 days of culturing on R2A agar plates. Gram staining, the hydrolysis assays for casein, starch and Tween 80 were performed according to the methods of Smibert and Krieg [32], and cellular morphology was observed using a transmission electron microscope (JEOL Ltd., Tokyo, Japan). Growth was assessed on Tryptic Soy Agar (TSA), R2A and PYG agar at 28℃. Growth was also tested at various temperatures (4–37℃), pH values (4.0–11.0) and NaCl concentrations (0–3.5%, at 0.5% intervals) in PYG medium. Carbon source utilization was assessed with API ID 32 GN strips (bioMérieux, France), and physiological and biochemical properties were evaluated using API 20NE, API 20E and API ZYM strips (bioMérieux) following the manufacturer’s instructions. Oxidase activity was tested using 3% (v/v) H_2_O_2_, and cytochrome oxidase activity was evaluated using 1% (w/v) tetramethyl-p-phenylenediamine. Fatty acid composition was analysed by harvesting cells from colonies grown on R2A agar after incubation at 28 °C. Fatty acid extraction was performed according to the MIDI 6.0 protocol [33]. The samples were analysed using an Agilent 6890 N gas chromatography system (Agilent Technologies, Santa Clara, CA, USA) equipped with an HP-ULTRA two-capillary column (25 m×0.2 mm×0.33 µm; Agilent). Identification of fatty acids was based on the TSBA6 database (Sherlock version 6.0B).

All eight representative strains were Gram-stain-negative, aerobic and rod-shaped (Fig. S3). They grew on TSA, PYG and R2A media, except J22R133^T^, which did not grow on TSA. Growth occurred at a temperature range of 10–30 °C, with J22P271^T^, J22R187^T^ and J22R193^T^ tolerating 4–35 °C or 4–30 °C, suggesting cold adaptation. Strain J22P271^T^ exhibits the broadest pH range, from 4 to 11. NaCl tolerance varied from 0–0.5% to 0–2.5%, with J2L1-78^T^ and J22P271^T^ showing higher tolerance (0–2.0% and 0–2.5%). Nitrate reduction and the Voges–Proskauer test were positive in J2P1-59^T^, J22R24^T^, J22P168^T^, J22P271^T^ and J22R193^T^, but negative in J2L1-78^T^, J22R133^T^ and J22R187^T^. Citrate utilization was positive in J2L1-78^T^, J22R24^T^ and J22P168^T^, but absent in others. All strains except strain J22R133^T^ possessed a flagellum. The differential phenotypic traits of these strains were presented in Table 1. Fatty acid profiles showed C_16 : 0_ (26.9–36.4%), summed feature 3 (C_16 : 1_* ω*7c and/or C_16 : 1_* ω*6c; 17.5–29.2%) and C_17 : 0_ cyclo (10.0–21.2%) as major components (Table S5). Summed feature 8 (C_18 : 1_* ω*6*c* and/or C_18 : 1_* ω*7*c*; 9.1–15.2%) was also prominent. Minor fatty acids, such as C_12 : 0_ 2-OH, were unique to strain J2L1-78^T^ (2.5%), while C_18 : 0_ was elevated in J22R187^T^ (6.9%) compared to 0.4–1.5% in others. These fatty acid profiles align with those of other *Variovorax* species [34].

**Table 1.** Differential phenotypic characteristics of strains J2L1-78^T^, J2P1-59^T^, J22R24^T^, J22R133^T^, J22P168^T^, J22P271^T^, J22R187^T^ and J22R193^T^

Characteristic	1	2	3	4	5	6	7	8
Growth temperature range (℃)	10–35	10–35	10–30	10–30	10–30	4–35	4–35	4–30
pH range for growth	5–10	5–11	5–9	5–9	5–10	4–11	5–10	5–10
NaCl range for growth (w/v, %)	0–2.0	0–1.5	0–1.0	0–0.5	0–1.5	0–2.5	0–1.5	0–1.0
Reduction of nitrate to nitrite	−	+	+	−	+	+	−	+
Voges–Proskauer test	−	+	+	−	+	+	−	+
Citrate utilization	+	−	+	+	−	+	−	−
**Enzyme activity:**								
Arginine dihydrolase	+	+	+	+	−	+	+	+
Lysine decarboxylase	+	−	−	+	−	+	−	+
Ornithine decarboxylase	+	−	−	−	−	+	−	+
Urease	+	+	+	+	−	+	+	+
Alkaline phosphatase	−	+	+	+	+	+	+	+
Lipase (C14)	−	+	+	+	−	−	+	+
Valine arylamidase	−	+	+	+	−	−	+	+
Cystine arylamidase	−	+	+	−	−	−	+	+
Acid phosphatase	+	+	+	+	+	+	−	+
*β*-Galactosidase	+	−	−	−	−	−	−	−
**Utilization of:**								
d-Mannitol	−	+	−	+	−	+	+	+
d-Glucose	−	+	+	+	−	+	+	+
d-Sorbitol	+	−	+	−	−	−	−	+
Propionate	−	+	+	+	+	+	+	+
Caprate	−	+	+	−	+	+	−	−
Valerate	+	+	+	+	+	+	+	+
Histidine	+	+	+	+	+	+	−	+
3-Hydroxybutyrate	+	+	+	+	+	−	+	+
4-Hydroxybenzoate	−	+	+	−	+	+	+	+
d-Ribose	−	−	+	−	−	−	−	+
Sucrose	−	−	−	−	−	−	−	+
Itaconate	−	−	+	+	−	−	−	+
Malonate	+	+	−	−	+	+	+	−
Lactate	+	+	+	+	+	+	−	+
l-Alanine	−	+	+	−	+	+	−	+
5-Ketogluconate	+	+	−	−	+	+	−	+
Glycogen	−	−	−	−	−	−	−	+
3-Hydroxybenzoate	+	+	+	+	−	+	+	+
l-Serine	−	−	−	−	−	−	−	+

1, J2L1-78T; 2. J2P1-59T; 3, J22R24T; 4, J22R133T; 5, J22P168T; 6, J22P271T; 7, J22R187T; 8, J22R193T. +, positive; −, negative.

Based on a comprehensive analysis encompassing phylogenetic, physiological, chemotaxonomic and genotypic data, the 13 strains (J2L1-78^T^, J2P1-59^T^, J22R24^T^, J22R133^T^, J22P168^T^, J22P271^T^, J22R187^T^, J22R193^T^, J2L1-63, J2R1-6, J22G21, J22G47 and J31P216) represent 8 novel *Variovorax* species. We propose the following names: *Variovorax arabinosiphilus* sp. nov. (type strain=J2 L1-78^T^), *Variovorax flavidus* sp. nov. (type strain=J2 P1-59^T^), *Variovorax gracilis* sp. nov. (type strain=J22 R24^T^), *Variovorax brevis* sp. nov. (type strain=J22 R133^T^), *Variovorax jilinensis* sp. nov. (type strain=J22 P168^T^), *Variovorax davisae* sp. nov. (type strain=J22 P271^T^), *Variovorax saccharolyticus* sp. nov. (type strain=J22 R187^T^) and *Variovorax fucosicus* sp. nov. (type strain=J22 R193^T^). This discovery, stemming from the rhizosphere of 20-year-old ginseng plants in Northeast China, significantly enhances our understanding of *Variovorax* diversity and its ecological role within plant microbiomes. Utilizing a polyphasic taxonomic approach that integrates 16S rRNA gene sequencing, whole-genome analysis and physiological profiling, we have confirmed the distinctiveness of eight type strains. A phylogenomic tree, constructed from 81 core genes and supplemented with 76 related genomic sequences, delineated 99 species across 9 clades, with these strains prominently positioned in clusters 4, 5, 6 and 7, revealing previously unrecognized diversity. The presence of *acdS* and *aldA* genes in several strains underscores their plant growth-promoting potential via ACC deaminase and IAA production, aligning with *Variovorax*’s role in mitigating root growth inhibition. The addition of eight novel species increases the total number of validly published *Variovorax* species to 21. This study not only highlights the extraordinary microbial diversity of the rhizosphere but also emphasizes the agricultural potential of *Variovorax*, particularly for ginseng cultivation. Further investigation into their physiological functions and adaptive mechanisms is essential to deepen our understanding of their ecological importance.

## Protologues

### Description of *Variovorax arabinosiphilus* sp. nov**.**

*Variovorax arabinosiphilus* (a.ra.bi.no.si'phi.lus. N.L. neut. n. *arabinosum*, arabinose; N.L. masc. adj. *philus*, loving; N.L. masc. adj. *arabinosiphilus*, arabinose-loving).

Cells are Gram-stain-negative, aerobic, rod-shaped and motile with a single flagellum, measuring 0.5–0.6 µm×1.2–2.6 µm. Colonies are yellow, convex and round, ~1.0 mm in diameter after 2 days of incubation on R2A plates at 28 °C. Growth occurs at temperatures between 10 and 35 °C (optimum 28 °C), at pH 5.0–10.0 (optimum pH 7.0), at 0–2.0% (w/v) NaCl and on PYG, TSA and R2A media. Positive for oxidase and catalase. Hydrolyzes starch and aesculin, but not casein, gelatin or Tween 80. Indole and H_2_S are not produced. Positive for citrate utilization, arginine dihydrolase, lysine decarboxylase, ornithine decarboxylase, urease, tryptophan deaminase, esterase (C4), esterase lipase (C8), leucine arylamidase, acid phosphatase, naphthol-AS-BI-phosphohydrolase and *β*-galactosidase. Negative for reduction of nitrate to nitrite, fermentation of glucose, Voges–Proskauer test, alkaline phosphatase, lipase (C14), valine arylamidase, cystine arylamidase, trypsin, *α*-chymotrypsin, *α*-galactosidase, *β*-glucuronidase, *α*-glucosidase, *β*-glucosidase, *N*-acetyl-*β*-glucosaminidase, *α*-mannosidase and *α*-fucosidase. Utilize the following carbon sources: l-arabinose, d-sorbitol, valerate, citrate, histidine, 2-ketogluconate, 3-hydroxybutyrate, l-proline, suberate, malonate, acetate, lactate, 5-ketogluconate and 3-hydroxybenzoate. Acids are produced from l-arabinose. Acids are not produced from amygdalin, d-mannitol, melibiose, d-sorbitol, sucrose, glucose, inositol and rhamnose. The major fatty acids (>10%) include C_16 : 0_, summed feature 3 (C_16 : 1_* ω*7c and/or C_16 : 1_* ω*6c) and C_17 : 0_ cyclo. The DNA G+C content of the type strain is 67.9 mol%.

The type strain, J2L1-78^T^ (=CGMCC 1.60704^T^=KACC 23365^T^), was isolated from the ginseng rhizosphere in Tonghua, Jilin Province, P.R. China. J2L1-63 and J2R1-6 are the second and third strains of the species. The NCBI accession numbers for the 16S rRNA gene and genome sequences are OR144162 and JASZYB000000000, respectively.

### Description of *Variovorax flavidus* sp. nov.

*Variovorax flavidus* (fla’vi.dus. L. masc. adj. *flavidus*, yellowish).

Cells are Gram-stain-negative, aerobic, rod-shaped and motile with a single flagellum, measuring 0.6–0.9 µm×1.1–2.0 µm. Colonies are yellow, convex and round, ~1.0 mm in diameter after 2 days of incubation on R2A plates at 28 °C. Growth occurs at temperatures between 10 and 35 °C (optimum 28 °C), at pH 5.0–10.0 (optimum pH 7.0), at 0–1.5% (w/v) NaCl and on PYG, TSA and R2A media. Positive for oxidase and catalase. Hydrolyzes starch and aesculin, but not casein, gelatin or Tween 80. Indole and H_2_S are not produced. Positive for reduction of nitrate to nitrite, Voges–Proskauer test, arginine dihydrolase, urease, alkaline phosphatase, esterase (C4), esterase lipase (C8), lipase (C14), leucine arylamidase, valine arylamidase, cystine arylamidase, acid phosphatase and naphthol-AS-BI-phosphohydrolase. Negative for fermentation of glucose, citrate utilization, lysine decarboxylase, ornithine decarboxylase, tryptophan deaminase, trypsin, *α*-chymotrypsin, *α*-galactosidase, *β*-galactosidase, *β*-glucuronidase, *α*-glucosidase, *β*-glucosidase, *N*-acetyl-*β*-glucosaminidase, *α*-mannosidase and *α*-fucosidase. Utilize the following carbon sources: d-mannitol, d-glucose, propionate, caprate, valerate, histidine, 2-ketogluconate, 3-hydroxybutyrate, 4-hydroxybenzoate, l-proline, suberate, malonate, acetate, lactate, l-alanine, 5-ketogluconate and 3-hydroxybenzoate. Acids are not produced from amygdalin, d-mannitol, melibiose, d-sorbitol, sucrose, glucose, inositol, l-arabinose and rhamnose. The major fatty acids (>10%) include C_16 : 0_, summed feature 3 (C_16 : 1_* ω*7c and/or C_16 : 1_* ω*6c), C_17 : 0_ cyclo and summed feature 8 (C_18 : 1_* ω*6*c* and/or C_18 : 1_* ω*7*c*). The DNA G+C content of the type strain is 65.9 mol%.

The type strain, J2P1-59^T^ (=CGMCC 1.60707^T^=KACC 23366^T^), was isolated from the ginseng rhizosphere in Tonghua, Jilin Province, P.R. China. The NCBI accession numbers for the 16S rRNA gene and genome sequences are OR144165 and JASZYE000000000, respectively.

### Description of *Variovorax gracilis* sp. nov.

*Variovorax gracilis* (gra'ci.lis. L. masc. adj. *gracilis*, slender, elongated, meant to denote slender, elongated cells).

Cells are Gram-stain-negative, aerobic, rod-shaped and motile with a single flagellum, measuring 0.6–0.7 µm×1.5–2.7 µm. Colonies are yellow, convex and round, ~1.0 mm in diameter after 2 days of incubation on R2A plates at 28 °C. Growth occurs at temperatures between 10 and 30 °C (optimum 28 °C), at pH 5.0–9.0 (optimum pH 7.0), at 0–1.0% (w/v) NaCl and on PYG, TSA and R2A media. Positive for oxidase and catalase. Hydrolyzes starch and aesculin, but not casein, gelatin or Tween 80. Indole and H_2_S are not produced. Positive for reduction of nitrate to nitrite, Voges–Proskauer test, citrate utilization, arginine dihydrolase, urease, alkaline phosphatase, esterase (C4), esterase lipase (C8), lipase (C14), leucine arylamidase, valine arylamidase, cystine arylamidase, acid phosphatase and naphthol-AS-BI-phosphohydrolase. Negative for fermentation of glucose, lysine decarboxylase, ornithine decarboxylase, tryptophan deaminase, trypsin, *α*-chymotrypsin, *α*-galactosidase, *β*-galactosidase, *β*-glucuronidase, *α*-glucosidase, *β*-glucosidase, *N*-acetyl-*β*-glucosaminidase, *α*-mannosidase and *α*-fucosidase. Utilize the following carbon sources: d-glucose, d-sorbitol, propionate, caprate, valerate, histidine, 2-ketogluconate, 3-hydroxybutyrate, 4-hydroxybenzoate, l-proline, d-ribose, itaconate, suberate, acetate, lactate, l-alanine and 3-hydroxybenzoate. Acids are not produced from amygdalin, d-mannitol, melibiose, d-sorbitol, sucrose, glucose, inositol, l-arabinose and rhamnose. The major fatty acids (>10%) include summed feature 3 (C_16 : 1_* ω*7c and/or C_16 : 1_* ω*6c), C_16 : 0_, summed feature 8 (C_18 : 1_* ω*6*c* and/or C_18 : 1_* ω*7*c*) and C_17 : 0_ cyclo. The DNA G+C content of the type strain is 65.8 mol%.

The type strain, J22R24^T^ (=CGMCC 1.61001^T^=KACC 23367^T^), was isolated from the ginseng rhizosphere in Tonghua, Jilin Province, P.R. China. The NCBI accession numbers for the 16S rRNA gene and genome sequences are OR144166 and JASZYF000000000, respectively.

### Description of *Variovorax brevis* sp. nov.

*Variovorax brevis* (bre'vis. L. masc. adj. *brevis*, short).

Cells are Gram-stain-negative, aerobic, rod-shaped and non-motile, measuring 1.1–1.3 µm×1.4–2.0 µm. Colonies are yellow, convex and round, ~1.0 mm in diameter after 2 days of incubation on R2A plates at 28 °C. Growth occurs at temperatures between 10 and 30 °C (optimum 28 °C), at pH 5.0–9.0 (optimum pH 7.0), at 0–0.5% (w/v) NaCl and on PYG and R2A media. Positive for oxidase and catalase. Hydrolyzes starch and aesculin, but not casein, gelatin or Tween 80. Indole and H_2_S are not produced. Positive for citrate utilization, arginine dihydrolase, lysine decarboxylase, urease, alkaline phosphatase, esterase (C4), esterase lipase (C8), lipase (C14), leucine arylamidase, valine arylamidase, acid phosphatase and naphthol-AS-BI-phosphohydrolase. Negative for reduction of nitrate to nitrite, fermentation of glucose, Voges–Proskauer test, ornithine decarboxylase, tryptophan deaminase, cystine arylamidase, trypsin, *α*-chymotrypsin, *α*-galactosidase, *β*-galactosidase, *β*-glucuronidase, *α*-glucosidase, *β*-glucosidase, *N*-acetyl-*β*-glucosaminidase, *α*-mannosidase and *α*-fucosidase. Utilize the following carbon sources: d-mannitol, d-glucose, propionate, valerate, histidine, 2-ketogluconate, 3-hydroxybutyrate, l-proline, itaconate, suberate, acetate, lactate and 3-hydroxybenzoate. Acids are not produced from amygdalin, d-mannitol, melibiose, d-sorbitol, sucrose, glucose, inositol, l-arabinose and rhamnose. The major fatty acids (>10%) include C_16 : 0_, summed feature 3 (C_16 : 1_* ω*7c and/or C_16 : 1_* ω*6c), C_17 : 0_ cyclo and summed feature 8 (C_18 : 1_* ω*6*c* and/or C_18 : 1_* ω*7*c*). The DNA G+C content of the type strain is 64.8 mol%.

The type strain, J22R133^T^ (=CGMCC 1.61263^T^=KACC 23368^T^), was isolated from the ginseng rhizosphere in Tonghua, Jilin Province, P.R. China. The NCBI accession numbers for the 16S rRNA gene and genome sequences are OR144167 and JASZYG000000000, respectively.

### Description of *Variovorax jilinensis* sp. nov.

*Variovorax jilinensis* (ji.lin.en’sis. N.L. masc. adj. *jilinensis*, pertaining to Jilin, a province in northeastern China, where the type strain was collected).

Cells are Gram-stain-negative, aerobic, rod-shaped and motile with a single flagellum, measuring 0.9–1.0 µm×1.6–2.0 µm. Colonies are yellow, convex and round, ~1.0 mm in diameter after 2 days of incubation on R2A plates at 28 °C. Growth occurs at temperatures between 10 and 30 °C (optimum 28 °C), at pH 5.0–10.0 (optimum pH 7.0), at 0–1.5% (w/v) NaCl and on PYG, TSA and R2A media. Positive for oxidase and catalase. Hydrolyzes starch and aesculin, but not casein, gelatin, or Tween 80. Indole and H_2_S are not produced. Positive for reduction of nitrate to nitrite, Voges–Proskauer test, alkaline phosphatase, esterase (C4), esterase lipase (C8), leucine arylamidase, acid phosphatase and naphthol-AS-BI-phosphohydrolase. Negative for fermentation of glucose, citrate utilization, arginine dihydrolase, lysine decarboxylase, ornithine decarboxylase, urease, tryptophan deaminase, lipase (C14), valine arylamidase, cystine arylamidase, trypsin, *α*-chymotrypsin, *α*-galactosidase, *β*-galactosidase, *β*-glucuronidase, *α*-glucosidase, *β*-glucosidase, *N*-acetyl-*β*-glucosaminidase, *α*-mannosidase and *α*-fucosidase. Utilize the following carbon sources: l-arabinose, propionate, caprate, valerate, histidine, 2-ketogluconate, 3-hydroxybutyrate, 4-hydroxybenzoate, l-proline, suberate, malonate, acetate, lactate, l-alanine and 5-ketogluconate. Acids are not produced from amygdalin, d-mannitol, melibiose, d-sorbitol, sucrose, glucose, inositol, l-arabinose and rhamnose. The major fatty acids (>10%) include C_16 : 0_, C_17 : 0_ cyclo, summed feature 3 (C_16 : 1_* ω*7c and/or C_16 : 1_* ω*6c) and summed feature 8 (C_18 : 1_* ω*6*c* and/or C_18 : 1_* ω*7*c*). The DNA G+C content of the type strain is 68.0 mol%.

The type strain, J22P168^T^ (=CGMCC 1.64555^T^=KACC 23372^T^), was isolated from the ginseng rhizosphere in Tonghua, Jilin Province, P.R. China. The NCBI accession numbers for the 16S rRNA gene and genome sequences are OR144177 and JASZYQ000000000, respectively.

### Description of *Variovorax davisae* sp. nov.

*Variovorax davisae* (da.vi’sae. N.L. gen. n. *davisae*, named in honour of Diana H. Davis, who discovered the first species of the genus *Variovorax*).

Cells are Gram-stain-negative, aerobic, rod-shaped and motile with a single flagellum, measuring 0.8–0.9 µm×1.5–2.4 µm. Colonies are yellow, convex and round, ~1.0 mm in diameter after 2 days of incubation on R2A plates at 28 °C. Growth occurs at temperatures between 4 and 35 °C (optimum 28 °C), at pH 4.0–11.0 (optimum pH 7.0), at 0–2.5% (w/v) NaCl and on PYG, TSA and R2A media. Positive for oxidase and catalase. Hydrolyzes starch and aesculin, but not casein, gelatin, or Tween 80. Indole and H_2_S are not produced. Positive for reduction of nitrate to nitrite, Voges–Proskauer test, citrate utilization, arginine dihydrolase, lysine decarboxylase, ornithine decarboxylase, urease, alkaline phosphatase, esterase (C4), esterase lipase (C8), leucine arylamidase, acid phosphatase and naphthol-AS-BI-phosphohydrolase. Negative for fermentation of glucose, tryptophan deaminase, lipase (C14), valine arylamidase, cystine arylamidase, trypsin, *α*-chymotrypsin, *α*-galactosidase, *β*-galactosidase, *β*-glucuronidase, *α*-glucosidase, *β*-glucosidase, *N*-acetyl-*β*-glucosaminidase, *α*-mannosidase and *α*-fucosidase. Utilize the following carbon sources: d-mannitol, d-glucose, l-arabinose, propionate, caprate, valerate, citrate, histidine, 2-ketogluconate, 4-hydroxybenzoate, l-proline, suberate, malonate, acetate, lactate, l-alanine, 5-ketogluconate and 3-hydroxybenzoate. Acids are not produced from amygdalin, d-mannitol, melibiose, d-sorbitol, sucrose, glucose, inositol, l-arabinose and rhamnose. The major fatty acids (>10%) include C_16 : 0_, summed feature 3 (C_16 : 1_* ω*7c and/or C_16 : 1_* ω*6c), summed feature 8 (C_18 : 1_* ω*6*c* and/or C_18 : 1_* ω*7*c*) and C_17 : 0_ cyclo. The DNA G+C content of the type strain is 68.2 mol%.

The type strain, J22P271^T^ (=CGMCC 1.64593^T^=KACC 23373^T^), was isolated from the ginseng rhizosphere in Tonghua, Jilin Province, P.R. China. The NCBI accession numbers for the 16S rRNA gene and genome sequences are OR144179 and JASZYS000000000, respectively.

### Description of *Variovorax saccharolyticus* sp. nov.

*Variovorax saccharolyticus* (sac.cha.ro.ly’ti.cus. Gr. neut. n. *sakchar*, sugar; N.L. masc. adj. *lyticus*, (from Gr. masc. adj. *lytikos*), dissolving; N.L. masc. adj. *saccharolyticus*, lysing sugar).

Cells are Gram-stain-negative, aerobic, rod-shaped and motile with a single flagellum, measuring 0.8–0.9 µm×1.3–2.3 µm. Colonies are yellow, convex and round, ~1.0 mm in diameter after 2 days of incubation on R2A plates at 28 °C. Growth occurs at temperatures between 4 and 35 °C (optimum 28 °C), at pH 5.0–10.0 (optimum pH 7.0), at 0–1.5% (w/v) NaCl and on PYG, TSA and R2A media. Positive for oxidase and catalase. Hydrolyzes starch and aesculin, but not casein, gelatin, or Tween 80. Indole and H_2_S are not produced. Positive for arginine dihydrolase, urease, alkaline phosphatase, esterase (C4), esterase lipase (C8), lipase (C14), leucine arylamidase, valine arylamidase, cystine arylamidase, trypsin and naphthol-AS-BI-phosphohydrolase. Negative for reduction of nitrate to nitrite, fermentation of glucose, Voges–Proskauer test, citrate utilization, lysine decarboxylase, ornithine decarboxylase, tryptophan deaminase, *α*-chymotrypsin, acid phosphatase, *α*-galactosidase, *β*-galactosidase, *β*-glucuronidase, *α*-glucosidase, *β*-glucosidase, *N*-acetyl-*β*-glucosaminidase, *α*-mannosidase and *α*-fucosidase. Utilize the following carbon sources: d-mannitol, d-glucose, l-arabinose, propionate, valerate, 2-ketogluconate, 3-hydroxybutyrate, 4-hydroxybenzoate, l-proline, suberate, malonate, acetate and 3-hydroxybenzoate. Acids are not produced from amygdalin, d-mannitol, melibiose, d-sorbitol, sucrose, glucose, inositol, l-arabinose and rhamnose. The major fatty acids (>10%) include C_16 : 0_, summed feature 3 (C_16 : 1_* ω*7c and/or C_16 : 1_* ω*6c) and C_17 : 0_ cyclo. The DNA G+C content of the type strain is 68.0 mol%.

The type strain, J22R187^T^ (=CGMCC 1.64629^T^=KACC 23374^T^), was isolated from the ginseng rhizosphere in Tonghua, Jilin Province, P.R. China. J31P216 is the second strain of the species. The NCBI accession numbers for the 16S rRNA gene and genome sequences are OR144180 and JASZYT000000000, respectively.

### Description of *Variovorax fucosicus* sp. nov.

*Variovorax fucosicus* (fu.co’si.cus. N.L. masc. adj. *fucosicus*, pertaining to fucose).

Cells are Gram-stain-negative, aerobic, rod-shaped and motile with a single flagellum, measuring 0.7–0.8 µm×1.3–1.9 µm. Colonies are yellow, convex and round, ~1.0 mm in diameter after 2 days of incubation on R2A plates at 28 °C. Growth occurs at temperatures between 4 and 30 °C (optimum 28 °C), at pH 5.0–10.0 (optimum pH 7.0), at 0–1.0% (w/v) NaCl and on PYG, TSA and R2A media. Positive for oxidase and catalase. Hydrolyzes starch and aesculin, but not casein, gelatin, or Tween 80. Indole and H_2_S are not produced. Positive for reduction of nitrate to nitrite, Voges–Proskauer test, arginine dihydrolase, lysine decarboxylase, ornithine decarboxylase, urease, alkaline phosphatase, esterase (C4), esterase lipase (C8), lipase (C14), leucine arylamidase, valine arylamidase, cystine arylamidase, acid phosphatase and naphthol-AS-BI-phosphohydrolase. Negative for fermentation of glucose, citrate utilization, tryptophan deaminase, trypsin, *α*-chymotrypsin, *α*-galactosidase, *β*-galactosidase, *β*-glucuronidase, *α*-glucosidase, *β*-glucosidase, *N*-acetyl-*β*-glucosaminidase, *α*-mannosidase and *α*-fucosidase. Utilize the following carbon sources: d-mannitol, d-glucose, d-sorbitol, propionate, valerate, histidine, 2-ketogluconate, 3-hydroxybutyrate, 4-hydroxybenzoate, l-proline, d-ribose, sucrose, itaconate, suberate, acetate, lactate, l-alanine, 5-ketogluconate, glycogen, 3-hydroxybenzoate and l-serine. Acids are not produced from amygdalin, d-mannitol, melibiose, d-sorbitol, sucrose, glucose, inositol, l-arabinose and rhamnose. The major fatty acids (>10%) include C_16 : 0_, summed feature 3 (C_16 : 1_* ω*7c and/or C_16 : 1_* ω*6c), C_17 : 0_ cyclo and summed feature 8 (C_18 : 1_* ω*6*c* and/or C_18 : 1_* ω*7*c*). The DNA G+C content of the type strain is 65.4 mol%.

The type strain, J22R193^T^ (=CGMCC 1.64631^T^=KACC 23375^T^), was isolated from the ginseng rhizosphere in Tonghua, Jilin Province, P.R. China. J22G21 and J22G47 are the second and third strains of the species. The NCBI accession numbers for the 16S rRNA gene and genome sequences are OR144181 and JASZYU000000000, respectively.

## Supplementary material

10.1099/ijsem.0.006895 Uncited Supplementary Material 1.

## Abbreviations

ACC: 1-aminocyclopropane-1-carboxylate
ANI: average nucleotide identity
dDDH: digital DNA–DNA hybridization
IAA: indole-3-acetic acid
MAGs: metagenome-assembled genomes
ML: maximum-likelihood
MP: maximum-parsimony
NJ: neighbour-joining
PYG: peptone, yeast extract and glucose
R2A: Reasoner’s 2A agar
TSA: Tryptic Soy Agar
