# Supplementary material for: Variovorax arabinosiphilus sp. nov., Variovorax flavidus sp. nov., Variovorax gracilis sp. nov., Variovorax brevis sp. nov., Variovorax jilinensis sp. nov., Variovorax davisae sp. nov., Variovorax saccharolyticus sp. nov. and Variovorax fucosicus sp. nov., isolated from ginseng rhizosphere
Source: Int J Syst Evol Microbiol. 2025 Aug 29;75(8):006895. doi: 10.1099/ijsem.0.006895 (PMC12396923; doi:10.1099/ijsem.0.006895)
Supplement: Uncited Supplementary Material 1. [file ijsem-75-06895-s001.pdf]

Description of *Variovorax arabinosiphilus* sp. nov., *Variovorax flavidus* sp. nov., *Variovorax gracilis* sp. nov., *Variovorax brevis* sp. nov., *Variovorax jilinensis* sp. nov., *Variovorax davisae* sp. nov., *Variovorax saccharolyticus* sp. nov., and *Variovorax fucosicus* sp. nov., isolated from ginseng rhizosphere

Yu-Hang Jiang, Ce-Ce Yin, Lei-Lei Yang, Yu-Hua Xin, Qing Liu, and Jian Ye

International Journal of Systematic and Evolutionary Microbiology

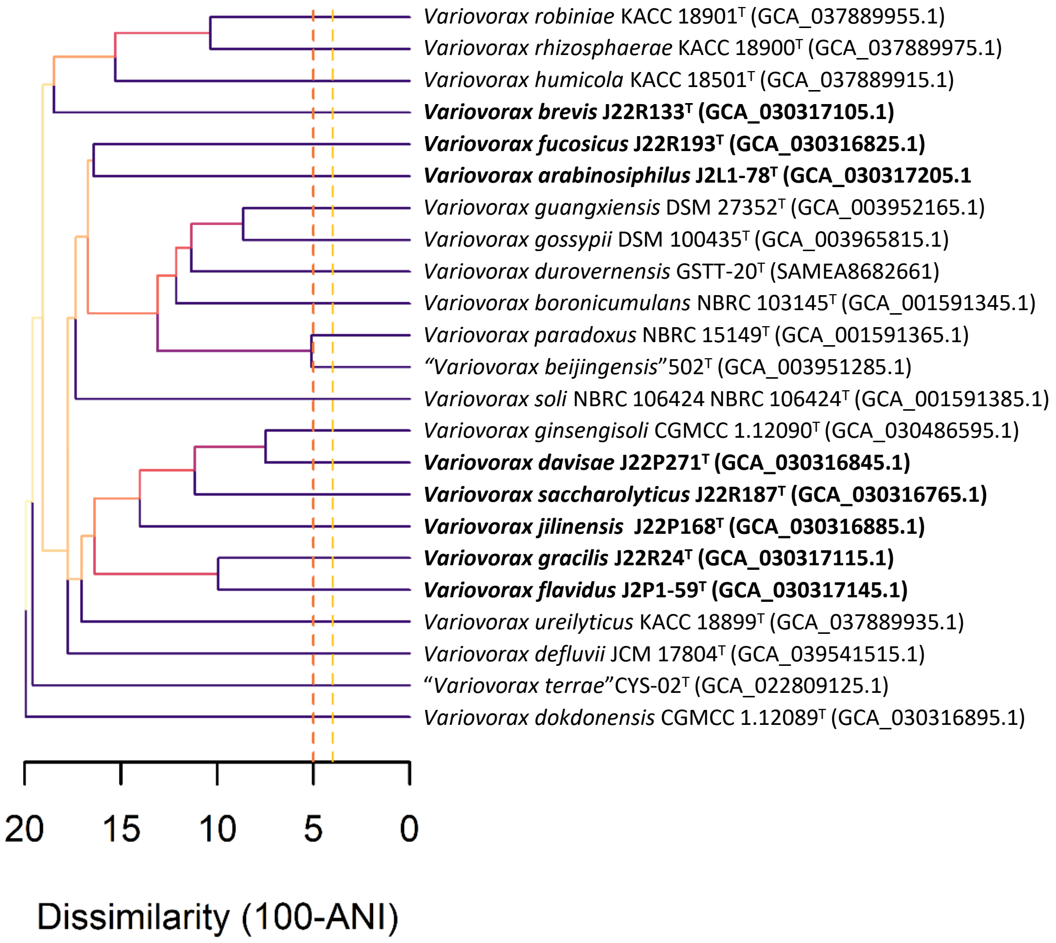

Fig. S1. Cluster analysis based on pairwise ANI values of the eight strains and related taxa. Accession numbers of the genomic sequences are provided in parentheses.

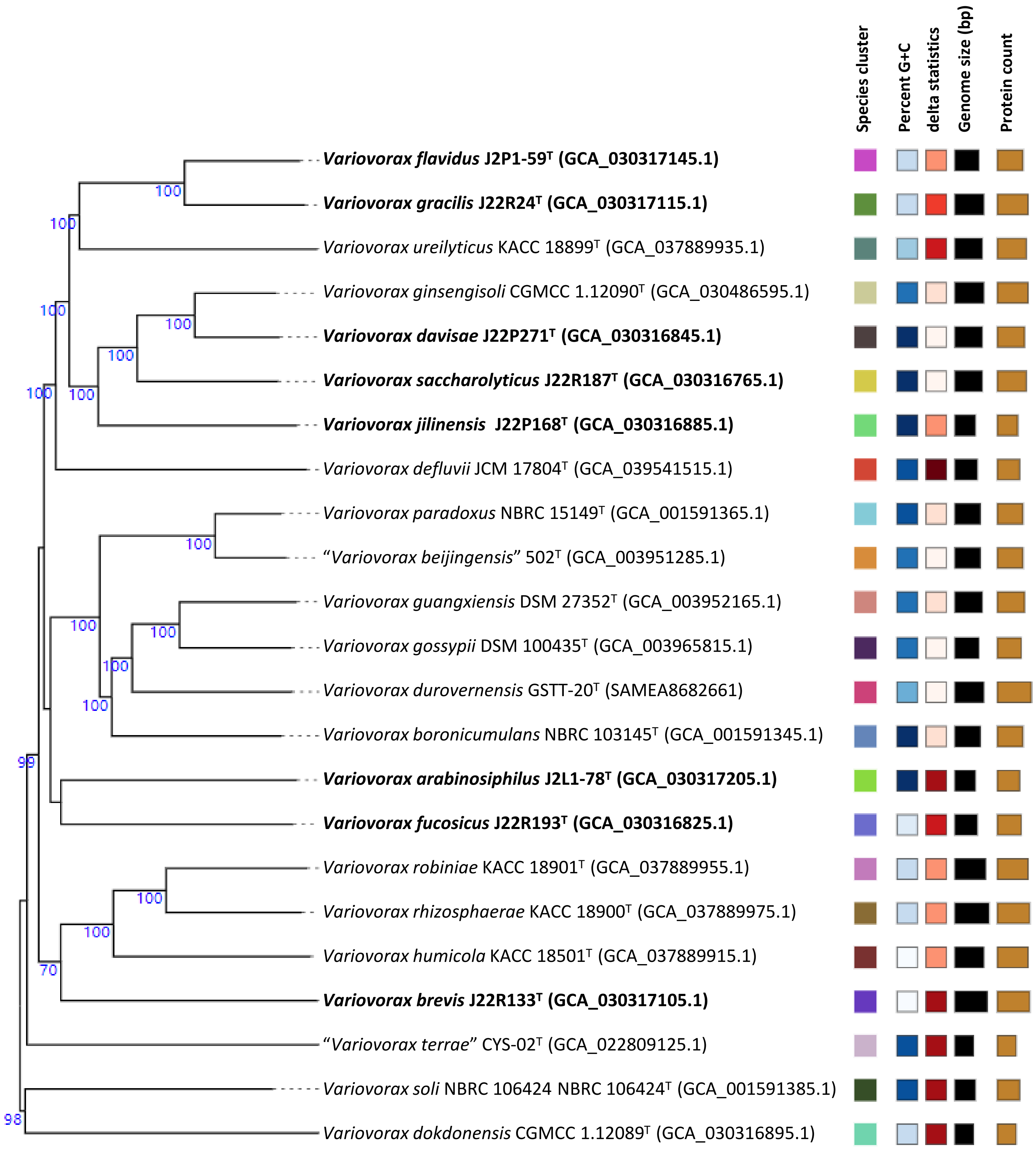

Fig. S2. Genome BLAST distance phylogeny (GBDP) of selected genomes, inferred using the TYGS webserver. Node values are based on 100 pseudo-bootstrap replicates.

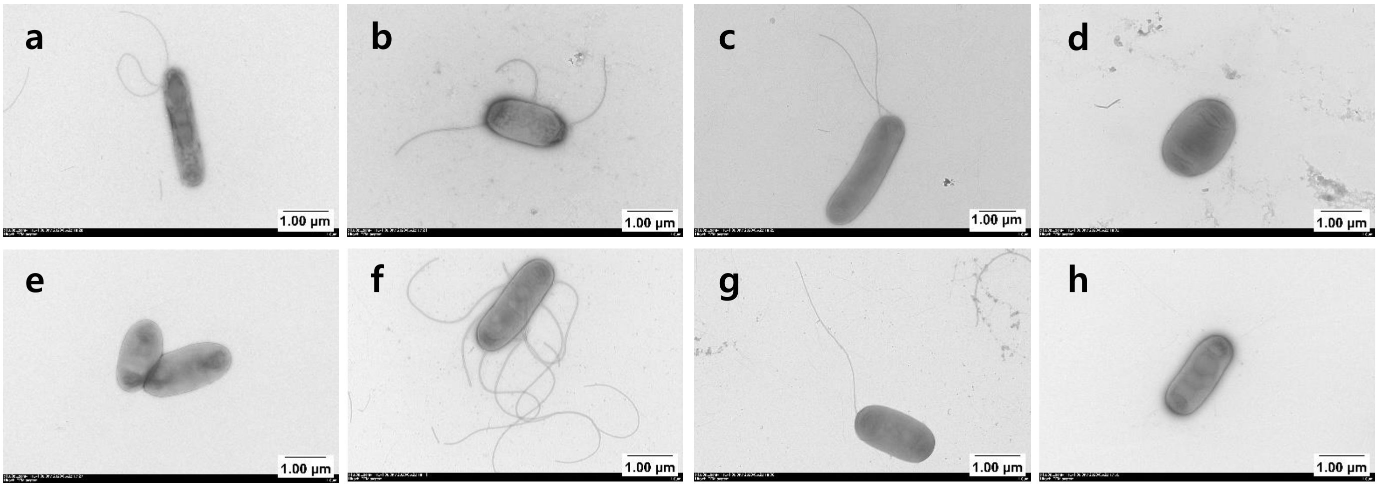

Fig. S3. Transmission electron micrograph of negatively stained cells of strains J2L1-78^{T} (a), J2P1-59^{T} (b), J22R24^{T} (c), J22R133^{T} (d), J22P168^{T} (e), J22P271^{T} (f), J22R187^{T} (g), and J22R193^{T} (h) grown at 28℃ on R2A agar. Scale bar, 1 µm.

Table S1. The basic information and the blast result using 16S rRNA gene sequences of the 13 strains.

Strain	CGMCC NO.	KACC NO.	16S rDNA accession no.	Closest relatives	16S rRNA gene sequence similarity(%)
**J2L1-78^{T}**	1.60704	23365	OR144162	*Variovorax ginsengisoli* Gsoil 3165^{T}	99.41
**J2P1-59^{T}**	1.60707	23366	OR144165	*Variovorax ureilyticus* UCM-2^{T}	99.34
**J22R24^{T}**	1.61001	23367	OR144166	*Variovorax ureilyticus* UCM-2^{T}	99.41
**J22R133^{T}**	1.61263	23368	OR144167	*Variovorax humicola* UC38^{T}	98.82
**J22P168^{T}**	1.64555	23372	OR144177	*Variovorax ginsengisoli* Gsoil 3165^{T}	99.56
**J22P271^{T}**	1.64593	23373	OR144179	*Variovorax ginsengisoli* Gsoil 3165^{T}	99.71
**J22R187^{T}**	1.64629	23374	OR144180	*Variovorax ginsengisoli* Gsoil 3165^{T}	99.85
**J22R193^{T}**	1.64631	23375	OR144181	*Variovorax ginsengisoli* Gsoil 3165^{T}	99.19
J2L1-63	1.60705		OR144163	*Variovorax ginsengisoli* Gsoil 3165^{T}	99.41
J2R1-6	1.60706		OR144164	*Variovorax ginsengisoli* Gsoil 3165^{T}	99.41
J22G21	1.63911		OR144168	*Variovorax ginsengisoli* Gsoil 3165^{T}	99.19
J22G47	1.63933		OR144170	*Variovorax ginsengisoli* Gsoil 3165^{T}	99.19
J31P216	1.64194		OR144175	*Variovorax ginsengisoli* Gsoil 3165^{T}	99.85

Table S2. Basic genome information for the 13 strains isolated in this study.

Strain	Proposal name	Genome accession no.	Genome size	GC (%)	Completeness (%)	Contamination (%)
**J2L1-78^{T}**	*Variovorax arabinosiphilus*	JASZYB000000000	6.11	67.9	100	0.65
**J2P1-59^{T}**	*Variovorax flavidus*	JASZYE000000000	8.33	65.9	100	0.94
**J22R24^{T}**	*Variovorax gracilis*	JASZYF000000000	7.74	65.8	100	0.52
**J22R133^{T}**	*Variovorax brevis*	JASZYG000000000	8.54	64.9	100	0.53
**J22P168^{T}**	*Variovorax jilinensis*	JASZYQ000000000	5.46	68.1	100	0.54
**J22P271^{T}**	*Variovorax davisae*	JASZYS000000000	7.15	68.3	100	0.53
**J22R187^{T}**	*Variovorax saccharolyticus*	JASZYT000000000	7.36	68.0	100	0.53
**J22R193^{T}**	*Variovorax fucosicus*	JASZYU000000000	6.11	65.4	100	2.38
J2L1-63	*Variovorax arabinosiphilus*	JASZYC000000000	5.65	67.9	100	0.83
J2R1-6	*Variovorax arabinosiphilus*	JASZYD000000000	5.65	67.9	100	0.53
J22G21	*Variovorax fucosicus*	JASZYH000000000	5.69	66.5	99.84	0.73
J22G47	*Variovorax fucosicus*	JASZYJ000000000	5.89	66.4	99.53	1.85
J31P216	*Variovorax saccharolyticus*	JASZYO000000000	8.14	67.5	100	0.47

Table S3. Genomic information for the 13 strains.

	Contigs	N50 (Mb)	gaps	CRISPR arrays	Coding density	CDSs	ncRNA regions	ncRNAs	rRNAs	tRNAs	tmRNAs	Hypotheticals	Pseudogenes
**J2L1-78^{T}**	9	2.89	0	0	92.8	5,294	17	6	6	49	1	282	2
**J2P1-59^{T}**	29	0.97	0	0	91.6	6,232	15	14	3	43	1	476	2
**J22R24^{T}**	104	0.27	0	0	90.0	7,280	18	9	3	45	1	745	11
**J22R133^{T}**	83	0.5	0	0	89.9	7,867	17	10	3	46	1	946	10
**J22P168^{T}**	21	0.86	0	0	92.5	5,126	13	6	6	47	1	326	2
**J22P271^{T}**	51	0.38	0	0	91.2	6,841	19	10	4	46	1	561	9
**J22R187^{T}**	25	0.49	0	0	91.5	6,932	19	9	4	52	1	470	4
**J22R193^{T}**	204	1.13	0	0	90.7	5,637	18	6	6	47	1	485	4
J2L1-63	11	1.42	0	0	92.8	5,296	17	6	6	49	1	399	2
J2R1-6	10	0.97	0	0	92.8	5,293	17	6	6	49	1	283	2
J22G21	18	1.27	0	0	93.0	5,325	17	6	6	46	1	234	4
J22G47	16	1.66	0	0	92.8	5,530	17	7	6	45	1	291	6
J31P216	68	0.27	0	0	90.1	7,636	19	11	3	53	1	823	4

Table S4. ANI values between the 13 strains isolated in this study.

	J2L1-78^{T}	J2L1-63	J2R1-6	J2P1-59^{T}	J22R24^{T}	J22R133^{T}	J22P168^{T}	J22P271^{T}	J22R187^{T}	J22R193^{T}	J22G21	J22G47
J2L1-63	**100.00**											
J2R1-6	**100.00**	**100.00**										
J2P1-59^{T}	82.89	82.83	82.95									
J22R24^{T}	82.64	82.68	82.66	90.86								
J22R133^{T}	81.88	81.91	81.92	81.92	82.05							
J22P168^{T}	84.07	84.01	84.07	84.63	84.21	81.83						
J22P271^{T}	84.00	83.96	84.09	85.10	84.70	81.85	87.32					
J22R187^{T}	83.89	83.87	83.93	85.17	84.82	81.88	87.36	89.73				
J22R193^{T}	84.41	84.35	84.48	82.83	82.50	81.95	83.83	83.99	83.79			
J22G21	84.36	84.30	84.38	82.88	82.48	81.98	83.93	83.97	83.85	**99.99**		
J22G47	84.23	84.14	84.22	82.84	82.48	81.83	83.79	83.78	83.75	**98.35**	**98.33**	
J31P216	83.81	83.75	83.82	85.20	84.78	81.90	87.25	89.89	**98.76**	83.77	83.77	83.66

Table S5. Cellular fatty acid composition of the eight proposed type strains.

Strains: 1, J2L1-78^{T}; 2. J2P1-59^{T}; 3, J22R24^{T}; 4, J22R133^{T}; 5, J22P168^{T}; 6, J22P271^{T}; 7, J22R187^{T}; 8, J22R193^{T}. Major fatty acids (>10 %) are represented in bold. TR, traces (<1 %); –, not detected. *Summed features represent fatty acids that cannot be resolved reliably from another fatty acid under the chosen chromatographic conditions. The MIDI system groups these fatty acids together as one feature with a single percentage of the total. Summed features consist of: 3, $C_{16:1}$ $\omega 7c$/$C_{16:1}$ $\omega 6c$; 5, $C_{18:0}$ ante/$C_{18:2}$ $\omega 6,9c$; 8, $C_{18:1}$ $\omega 6c$/$C_{18:1}$ $\omega 7c$.

Fatty acids	1	2	3	4	5	6	7	8
**Saturated**								
$C_{12:0}$	1.2	3.5	4.3	3.2	3.3	3.5	3.2	2.8
$C_{14:0}$	7.6	4.7	5.4	1.3	5.7	4.9	5.2	4.4
$C_{16:0}$	**31.6**	**26.9**	**28.7**	**36.4**	**33.6**	**32.3**	**31.6**	**31.1**
$C_{17:0}$	TR	TR	TR	-	TR	TR	TR	TR
$C_{18:0}$	TR	TR	TR	1.5	TR	TR	6.9	TR
$C_{17:0}$ cyclo	**11.5**	**21.2**	**10.0**	**12.7**	**19.8**	**14.8**	**10.2**	**15.4**
**Hydroxy**								
$C_{8:0}$ 3OH	-	-	-	-	-	-	1.2	TR
$C_{10:0}$ 3OH	3.2	4.3	6.1	4.8	4.9	3.7	4.3	3.4
$C_{12:0}$ 2OH	2.5	-	-	-	-	-	-	-
**Unsaturated**								
$C_{18:1}$ $\omega 9c$	-	-	-	-	-	-	3.0	-
**Summed feature***								
3	**29.2**	**18.7**	**29.1**	**23.0**	**17.5**	**21.2**	**19.6**	**24.1**
5	TR	TR	TR	1.6	TR	TR	-	TR
8	9.5	**15.2**	**13.8**	**11.2**	**12.6**	**17.1**	9.1	**14.9**
